# The current dengue outbreak amidst COVID-19 pandemic in Pakistan; a major threat to Pakistan’s healthcare system

**DOI:** 10.1016/j.amsu.2022.103670

**Published:** 2022-05-02

**Authors:** Shahzaib Ahmad, Fatima Yasin, Manas Pustake, Hadin Darain Khan, Inês F Silva Correia

**Affiliations:** a Mayo Hospital Lahore, Pakistan; b Grant Medical College and Sir JJ Group of Hospitals, Mumbai, India; c Shalamar Medical and Dental College Lahore, Pakistan; d School of Medicine, Faculty of HEMS, Anglia Ruskin University, Bishop Hall Lane, Chelmsford, CM1 1SQ, UK

Dear Editor,

Pakistan is facing another calamity in the form of a Dengue outbreak which has been surging since the advent of October 2021. Public and private hospitals in the federal capital Islamabad and other cities of Pakistan are under a great burden as this outbreak has struck amidst the COVID-19 pandemic. The situation has become alarming with the recent Dengue surge diverting attention from the COVID-19 pandemic and authorities failing to compile data, resulting in underreporting of cases. The immediate anti-dengue surveillance campaign has shown many hotspots where the Dengue vector is breeding continuously, and an anti-dengue campaign has been launched in the capital as well as in other provinces to cater to this issue. However, under-reporting and a deficiency in surveillance are aggravating the problem [1]. The target of this letter is to highlight this under-reporting and surveillance issue so that the proper interventions can be made for better protection of the population at risk from this lethal outbreak. Moreover, the global attraction to the problem is aimed at preventing its application elsewhere (see Table 1).

**Table 1:** Comparison of COVID-19 and dengue fever [[2], [3], [4], [5], [6], [7]].

	COVID-19	Dengue Fever
Clinical Features	Primarily cough and shortness of breath	Nausea, vomiting, rash, aches and pains (eye pain, typically behind the eyes, muscle, joint or bone pain)
Diagnosis	RT-PCR	NAAT and Serology
Prognosis	Variable	Poor
Risk Groups and Risk Factors	Close contact with infected person/Immunosuppression	Age >6 years, hepatomegaly, abdomen pain and oliguria were the most common risk factors associated with shock in children with Dengue fever
Treatment	Symptomatic (other drugs are under trial)	Under Trial
Vaccination	Undertrial (WHO has approved several for emergency use)	Under development

Dengue is an infectious disease caused by any of the four virus serotypes: DENVs [[2], [3], [4], [5]]. It is a mosquito-borne disease and is primarily transmitted to humans by the female Aedes mosquito. It is a daytime breeder, biting the victim early in the morning or just before dark and spreading quickly, with the victim developing a viremia after just 4 days of being bitten by an infected mosquito. The fever arises and subsides in a cyclical pattern and the victim's platelet count starts decreasing. The disease is mainly concentrated in tropical and subtropical regions, putting nearly a third of the human population, worldwide, at risk of infection. Infection with DENV results in varying degrees of pathological conditions, ranging from mild asymptomatic Dengue fever (DF) to severe Dengue hemorrhagic fever (DHF) and Dengue shock syndrome (DSS) which may be fatal. A dramatic worldwide expansion of the DENV has occurred due to rapid urbanisation, an increase in international travel, a lack of effective mosquito control measures and globalisation. Though there is no approved drug, an update by Sanofi Pasteur reveals licensing of its vaccine in Mexico, Brazil, the Philippines and El Salvador [3].

Dengue has been a problem for Pakistan for many years, having faced its first outbreak in 1994 and a sudden increase in human cases in October 2005. Dengue is now an epidemic in Pakistan and many outbreaks have been reported in 2010, 2017, 2019 and 2020. Cases of Dengue fever circulate throughout the year, with a peak incidence occurring in the monsoon season. The 2020 outbreak was amidst a COVID-19 pandemic that resulted in about 3,442 human cases of Dengue fever, leading to a significant threat to Pakistan's healthcare system. Furthermore, the country is now facing another dangerous outbreak of Dengue fever amidst the COVID-19 pandemic [8]. The National Institute of Health (NIH) of Islamabad, Pakistan, has published a report at the end of October 2021 in which it was said there were 25,478 cases of Dengue fever so far this year, with an escalation of almost 22,000 cases in just the past two months [1].

The challenges in combating the Dengue epidemic are many, including limited economic resources, lack of awareness, education, or action, limited community cohesion, limited sustainability of government interventions and an ineffective reporting system and surveillance. A complex combination of economic, environmental, health, political, and social factors can influence an individual's and community's adherence to suggested Dengue prevention strategies, either directly or indirectly [9]. *Pakistan's Emergency Plan of Action (EPoA): Dengue Response* is currently being enacted to curtail Dengue vector control. This project aims to limit the spread of vector transmission and develop prevention measures among vulnerable groups by providing long-lasting insecticidal nets (LLINs), mosquito repellent, spraying and fumigating mosquito breeding areas while raising community and educational awareness [10].

It is critical that the government investigates the problem before it escalates into a more dangerous situation for these communities, as well as committing adequate funding for preventative measures. Furthermore, an effective system for formal case reports should be in place so that the exact gravity of the situation can be determined quickly. By understanding how these intrinsic and extrinsic factors hinder adherence, health authorities can adopt national policies to strengthen community participatory action in vector control, empower leadership potential among health workers and community members and provide an appropriate and systemic approach to preventing disease transmission. A plan should be fully implemented, and the reaction to the program, as well as beneficiary input, should be analyzed and monitored. The public should be appropriately informed about current and previous epidemics so that they can play an important part in preventing future outbreaks, such as understanding how to seek medical treatment following a fever and avoid aspirin and ibuprofen if sick. Furthermore, the municipality should take proactive measures to keep public spaces and hospitals as clean as possible, while the community is taught on proper waste disposal and the dangers that can result if it is not done. Sources of stagnant water should also be discarded and avoided in order to eliminate the vector's reproductive grounds. All of these methods necessitate community involvement in order for a Dengue awareness program to function effectively through inter-sectoral coordination. The seriousness of the situation has been explained, and this letter is intended to draw the government's attention to the problem while it is still in its early stages. The impact on the health system is significant as the number of severe illnesses rises and more individuals require rapid hospitalization. To protect Pakistan's communities from a future outbreak, guidelines and effective control procedures should be implemented.

## Please state any sources of funding for your research

No funding

## Ethical approval

Not required.

## Consent

NA.

## Author contribution

Every author contributed to every stage of paper writing and design.

## Registration of research studies

Name of the registry:
Unique Identifying number or registration ID:
Hyperlink to your specific registration (must be publicly accessible and will be checked):

## Guarantor

Fatima Yasin

yasinfatima18@gmail.com.

## Declaration of competing interest

No conflict of interest.
